# Structural and Mechanical Properties of Konjac Glucomannan Gels and Influence of Freezing-Thawing Treatments on Them

**DOI:** 10.3390/polym14183703

**Published:** 2022-09-06

**Authors:** Hiroyuki Takeno, Ryuki Hashimoto, Yunqiao Lu, Wen-Chuan Hsieh

**Affiliations:** 1 Division of Molecular Science, Graduate School of Science and Technology, Gunma University, Kiryu 376-8515, Gunma, Japan; 2 Gunma University Center for Food Science and Wellness, 4-2 Aramaki, Maebashi 371-8510, Gunma, Japan; 3 Department of Biological Science and Technology, College of Medicine, I-SHOU University, No. 8, Yida, Yanchao, Kaohsiung 82445, Taiwan

**Keywords:** konjac glucomannan, freezing-thawing, synchrotron small-angle/wide-angle X-ray scattering

## Abstract

Freezing has been widely used for long-term food preservation. However, freezing-thawing (FT) treatment usually influences the texture and structure of food gels such as konjac. For their texture control after FT treatment, it is important to clarify the structural change of food gels during the FT process. In this study, we investigated the aggregated structures of konjac glucomannan (GM) gels during the FT process using simultaneous synchrotron small-angle X-ray/wide-angle X-ray scattering (SAXS/WAXS) techniques. The FT treatment resulted in more crystallization of GM, and consequently, a large increase in compressive stress. In-situ SAXS/WAXS measurements revealed the following findings: on freezing, water molecules came out of the aggregated phase of GM and after the thawing, they came back into the aggregated phase, but the aggregated structure did not return to the one before the freezing; the gel network enhanced the inhomogeneity due to the growth of ice crystals during freezing. Furthermore, we examined the influence of additives such as polyvinyl (alcohol) (PVA) and antifreeze glycoprotein (AFGP) on the mechanical and structural properties of freeze-thawed GM gels. Although the addition of PVA and AFGP suppressed the crystallization of GM, it could not prevent the growth of ice crystals and the increase in the inhomogeneity of the gel network. As a result, the compressive stresses for freeze-thawed GM gels containing PVA or AFGP were significantly higher compared with those of GM gels without FT treatments, although they were lower than those of freeze-thawed GM gels. The findings of this study may be useful for not only the texture control of freeze-thawed foods but also the improvement of the mechanical performance of the biomaterials.

## 1. Introduction

Konjac, which is made of mainly konjac flour and a large amount of water, is known as a food gel with a chewy texture. The major component of konjac flour is a polysaccharide, glucomannan (GM) [1,2], which is constituted by D-glucose and D-mannose units with partly acetyl groups in the backbone and a branched structure. GM gels are usually prepared by adding an alkali to GM aqueous solutions and subsequently heating them [3]. It is believed that the addition of alkali and heating induces the deacetylation and aggregation of GM to form a gel [3,4,5,6]. However, the aggregated structure of the GM gels remains poorly understood. Synchrotron small-angle X-ray scattering (SAXS) and wide-angle X-ray scattering (WAXS) are very powerful tools to examine structures of nanomaterials containing crystallites [7]. Simultaneous SAXS/WAXS measurements, in particular, allow us to pursue, in real-time, the structural change that is caused by the phase transition, self-assembly, and crystallization processes [8,9,10].

Freezing is a simple and effective method of food preservation; however, the texture of many foods is significantly affected by freezing-thawing (FT) treatment, which is a serious problem in the food industry [11]. Generally, ice crystals formed during storage at subzero temperatures damage the structure of foods. The texture of konjac glucomannan gels is greatly affected by the FT treatment as well as other foods [12,13]. Genevro et al. have reported that freezing rates affected the pore size of freeze-dried GM gels; a slow freezing rate (2.5 °C min^−1^) produced a structure with large pores (16.7 μm in length and 16.0 μm in width), whereas a rapid freezing rate (ca. 30 °C min^−1^) produced one with small pores (13.7 μm in length and 5.8 μm in width) [12]. In the field of material science, FT treatment has been used to improve the mechanical performance of materials. In particular, the mechanical and structural properties of freeze-thawed poly(vinyl alcohol) (PVA) and polysaccharide hydrogels have been investigated in depth [14,15,16,17,18].

Antifreeze proteins (AFP) and antifreeze glycoproteins (AFGP) have the characteristics of adsorbing on the surface of ice crystals so that they prevent ice crystal growth or ice recrystallization [19,20]. It has been reported that synthetic polymers such as PVA have antifreeze properties [21,22,23]. These materials may be useful to control the texture of foods after FT treatments. However, under the condition of frozen storage in a household freezer (~−20 °C), it is not clear how much the antifreeze materials are useful for the control of the microstructure and texture of food gels such as konjac. In the case of food gels, the effect of antifreeze materials on the texture of the freeze-thawed gels may be affected by the structure of the gel network or the gelation mechanism. Ishii and Inoue have examined the effect of antifreeze materials on the texture of various foods and food gels, and as a result, reported that the effect was different by the kinds of foods [24].

Thus, we investigated the structural and mechanical properties of GM hydrogels and the influence of FT treatment on them. For this purpose, using simultaneous synchrotron SAXS/WAXS techniques, we observed the structural change of GM gels during the FT process. Additionally, we examined the influence of the addition of AFGP or PVA on the structural and mechanical properties of the freeze-thawed GM gels.

## 2. Materials and Methods

### 2.1. Sample and Sample Preparation

Konjac GM (RHEOLEX RS) was kindly supplied from Simizu Chemical Corp, Mihara, Japan. In this study, we used sodium hydroxide (NaOH) as a solidifying agent, which was purchased from FUJIFILM Wako Pure Chemical Corp, Tokyo, Japan. As an additive, we used PVA with a molecular weight of 44,000 from Sigma-Aldrich (St. Louis, MO, USA), which was estimated from a viscosity measurement, and a fish-derived antifreeze glycoprotein (AFGP) with a molecular weight of 2600–33,000 from Nichirei. GM was dissolved in distilled water at 60 °C and afterward placed for 1 day. For samples containing additives, GM and PVA (or AFGP) were dissolved in distilled water in a water bath set at 60 °C, and then the GM/PVA (or GM/AFGP) aqueous solution was placed for 1 day. After a small amount of NaOH aqueous solution was added to the aqueous GM (or GM/PVA or GM/AFGP) solution to maintain it at pH = 11~12, the mixture was put in a given mold and heated in boiling distilled water. The final concentration of GM in the gels was 3% in weight.

### 2.2. Compression Measurements

Uniaxial compression measurements were performed at a compression speed of 10 mm/min using a compression test machine (AIKOH Engineering, Model-1305NR) with a Force Analyzer Explorer III and a load cell of 20 N for cylindrical GM gels with 14 mm in diameter and 8 mm in thickness. The measurements were conducted until the compressive force reached the maximum working load of the load cell. After the samples for the FT treatment were put in a freezer at −20 °C for a given period (1 h, 4 h, and 24 h), they were thawed at room temperature. Hereafter, we designate these samples FT 1 h, FT 4 h, and FT 24 h. Average values of compressive stresses at different strains were obtained from at least three measurements for each gel.

### 2.3. Synchrotron SAXS/WAXS Measurements

We conducted simultaneous synchrotron SAXS and WAXS measurements at beamline 6A at the photon factory in Tsukuba, Japan. The samples with 1 mm thickness were prepared for the measurements and then were put in a 1-mm thick spacer. Afterward, they were covered by two thin Kapton films. The X-ray beam with a wavelength of 1.5 Å was incident on the sample and the scattered X-ray was detected with two-dimensional detectors, PILATUS-1M for SAXS and PILATUS-100K for WAXS. The scattering images were circularly averaged with software [25] so that the scattering intensity was obtained as a function of the magnitude of the wavevector *q* defined by *q* = 4π sin(*θ*/2)/*λ*. Here *θ* and *λ* denote the scattering angle and the wavelength of the X-ray, respectively. After the obtained scattering profile was thus corrected for the background scattering intensity, the strength of the incident beam, and transmittance, the corrected intensity was calibrated in the absolute units using the glassy carbon [26]. SAXS/WAXS measurements during the FT process were conducted under the following conditions: the samples were cooled to −20 °C (−24 °C for GM/PVA gels) at a rate of 1 °C/min and heated to 10 °C at the same rate, and then heated to 25 °C using a temperature control system from Linkam Scientific Instruments Ltd.

### 2.4. Fourier Transform Infrared (FT-IR) Spectroscopy

Attenuated total reflection FT-IR measurements were performed using an FT-IR spectrometer (JASCO FT/IR 4700). The spectra were recorded in the range of wavenumbers from 500 to 4000 cm^−1^. The freeze-dried samples were used for the measurements.

### 2.5. Scanning Electron Microscopy Observation

The cross-section of freeze-dried samples was observed by a field emission scanning electron microscope (FE-SEM Hitachi-4700, Japan). The specimens for SEM observation were fixed on a metal disc with double-sided conductive adhesive tape and sputtering a thin platinum film on the sample. The FE-SEM observation was carried out under an accelerating voltage of 5 kV at a working distance (WD) of 12 mm. All samples were vacuumed for 24 h to completely remove the water before testing.

## 3. Results and Discussion

### 3.1. Structure of GM Gels

**Figure 1:**
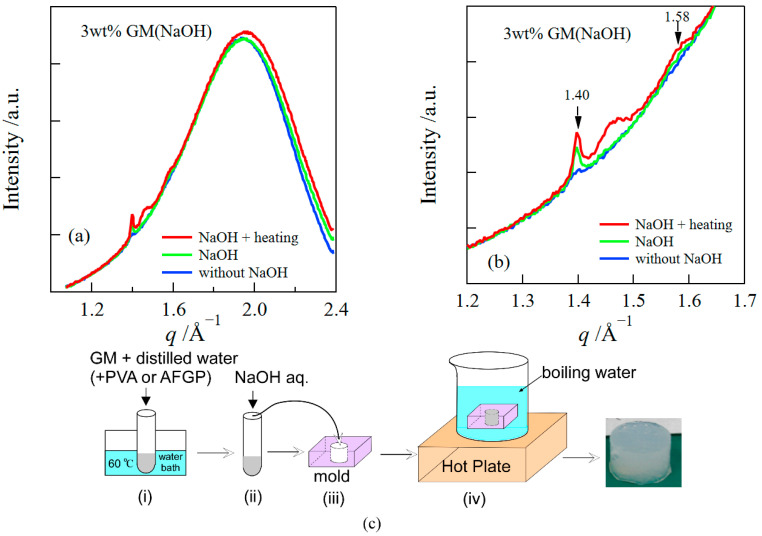
WAXS profiles of GM gels prepared in the various methods (“NaOH + heating”: (i)−(iv), “NaOH”: (i)−(iii), “without NaOH”: (i) + (iii)) (**a**). The magnification around the crystalline peaks (**b**). A flow diagram of the sample preparation (**c**).

Firstly, we examined the influence of the addition of NaOH and heating on the structure of GM gels (Figure 1). We show WAXS profiles (Figure 1a,b) for samples prepared in the various methods and a flow diagram of the sample preparation (Figure 1c). The broad and large peak observed at *q* ~ 1.95 Å^−1^ in WAXS profiles corresponds to the amorphous peak of water. Additionally, small peaks were observed at *q* ~ 1.40 and 1.58 Å^−1^, which were assigned to (200) and (220) reflections of mannan II (hydrated polymorph), respectively [27]. This behavior agreed with the result of the earlier studies, where it was reported that the crystal structure of deacetylated konjac GM is almost identical to that of mannan II [27,28]. Figure 1b presents the magnification of the WAXS curves around the crystalline peaks. The crystalline peak intensity increased by adding NaOH and heating, which suggested that alkali and heat treatments promoted the crystallization of GM.

**Figure 2:**
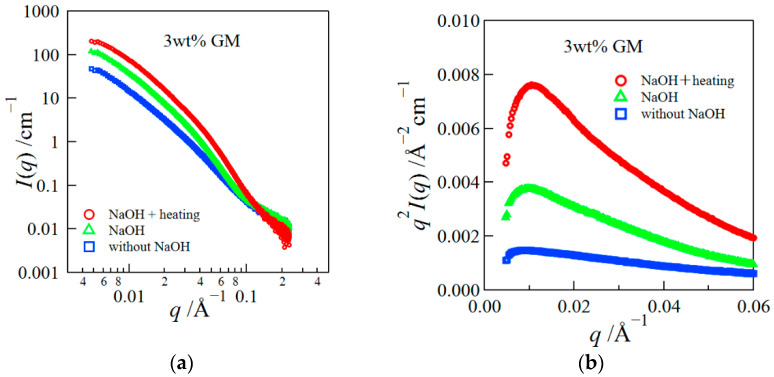
SAXS profiles for GM gels prepared in the various methods (**a**) and the Kratky plot (**b**).

Next, we show the SAXS result for the same samples (Figure 2). The SAXS intensity at small *q* largely increased by adding NaOH and heating, suggesting the aggregation of GM molecules. The Kracky plots for these samples had a definite peak for alkali- and heat-treated gels (Figure 2b), implying that the structural inhomogeneity increased due to the aggregation of GM molecules arising from the alkali and heat treatments [29].

**Figure 3:**
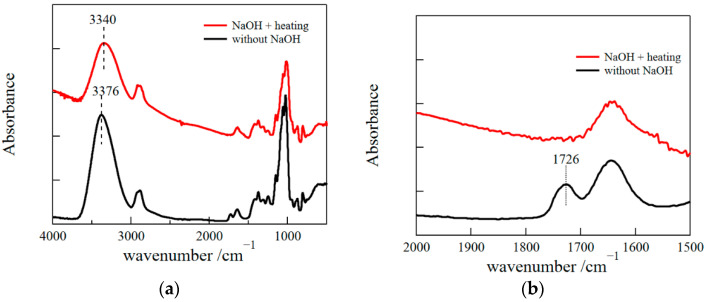
FTIR spectra for freeze-dried samples of GM gels prepared by adding NaOH and heating and for freeze-dried samples of GM aqueous solutions without NaOH (**a**) and the magnification (**b**).

Figure 3 depicts Fourier transform infrared (FT-IR) spectra for freeze-dried samples of a GM gel (solution) with and without alkali and heat treatments. The characteristic bands observed around 3300 cm^−1^ correspond to the stretching vibration of OH groups hydrogen-bonded between GM molecules. The band shifted to a lower wavenumber by adding NaOH and heating, suggesting a stronger hydrogen bond was formed due to the aggregation of GM.

### 3.2. Influence of FT Treatment on Mechanical and Structural Properties

**Figure 4:**
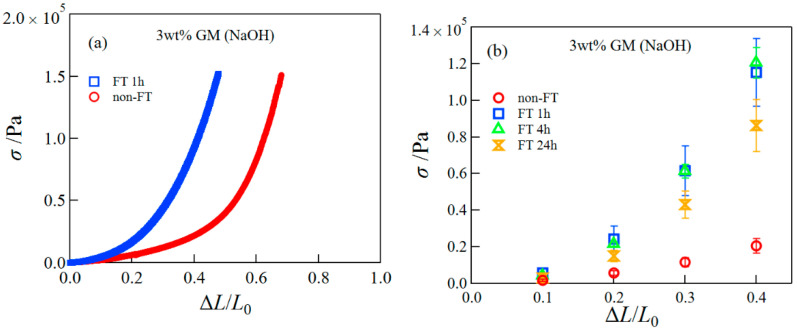

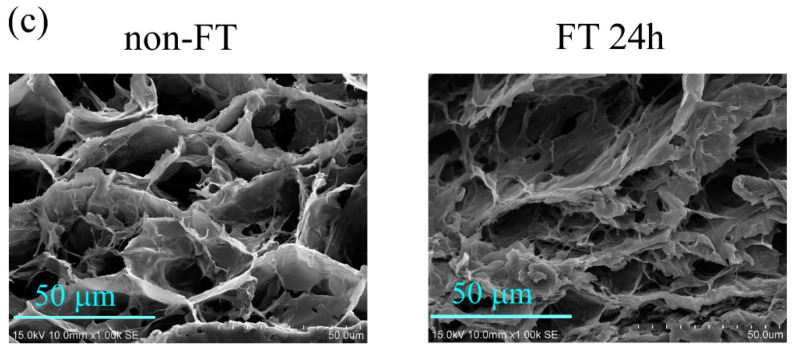
Representative compression stress–strain curves (**a**), the compressive stress at different strains (**b**), and SEM images of the cross-section of GM gels with and without FT treatment (**c**).

We examined the influence of the FT treatment on the mechanical properties of GM gels. Figure 4a presents representative compressive stress–strain curves for GM gels without and with the FT treatment (FT 1 h). The FT treatment largely enhanced the compressive stress, i.e., the GM gel after the FT treatment became significantly stiffer. Similar results were reported for GM gels using calcium hydroxide as an alkali and xanthan hydrogels [12,17]. Furthermore, we investigated the effect of the freezing period on the mechanical property of the GM gels (Figure 4b) The stresses were not influenced much by the freezing period. Next, we present SEM images of the cross-section of GM gels with and without FT treatment in Figure 4c. The SEM image of the GM gel without FT treatment showed the network structure with tens of μm of pore size, whereas the image of GM gel with FT treatment had a somewhat distorted network structure.

**Figure 5:**
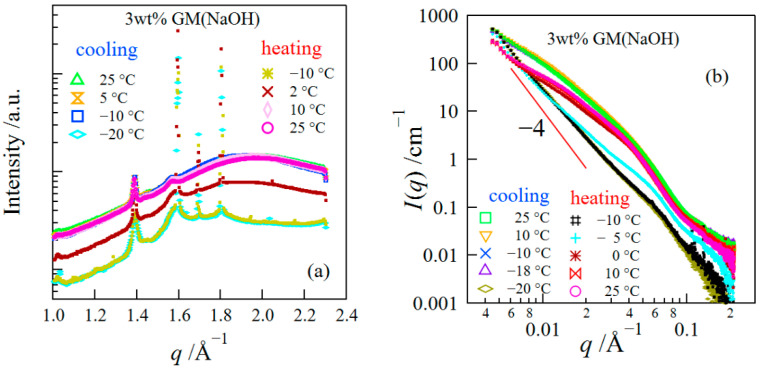
WAXS (**a**) and SAXS (**b**) curves for GM gels during the FT process.

We investigated the structural change of GM gels during the FT process using simultaneous SAXS/WAXS measurements. Figure 5a depicts the change in the WAXS profiles of GM gels during the FT process. The WAXS curves before freezing are similar to those shown in Figure 1, i.e., they are composed of a large broad peak from amorphous water and crystalline peaks of GM.

At −18 °C, very strong and sharp peaks appeared at 1.59, 1.69, and 1.80 Å^−1^, which were assigned to reflections from Ice Ih (hexagonal ice crystal) [30,31]. Afterward, as the temperature rose to temperatures above 0 °C, the crystalline peaks disappeared due to the fusion of ice crystals. In Figure 5b, we show the change in the SAXS curves during the FT process. The structures of GM gels during the FT process can be divided into three regions, (i) the structure before freezing, (ii) the structure during the freezing, and (iii) the structure after thawing. The structures of GM gels before freezing may be composed of the aggregated structure observed at low *q* (*q* < 0.1 Å^−1^) and the concentration fluctuations inside the aggregated phase observed at high *q* (*q* > 0.1 Å^−1^). The aggregation of GM triggered by adding alkali and heating led to an increase in the scattering intensity at *q* < 0.1 Å^−1^, whereas the scattering intensity at *q* > 0.1 Å^−1^ did not increase as shown in Figure 2a. When the hexagonal ice was formed, the SAXS curves drastically changed; the scattering curves showed the power–law behavior with an exponent of ~−4 at low *q*, whereas the scattering intensities at high *q* decreased. The latter result suggests that water came out of the aggregated phase so that the concentration fluctuations were suppressed. The large change in the scattering profiles due to the formation of ice crystals was also seen for other polymer hydrogels [32]. Besides, the SAXS profiles after thawing did not return to those before freezing. In this region, the scattering intensity at very low *q* (<0.0074 Å^−1^) increased upward, suggesting the inhomogeneity of the network was enhanced due to the FT treatment.

Here, we analyzed the SAXS data using the Beaucage equations composed of two structural levels [33,34]. This model well expresses the structure composed of multi-levels.

(1) $$I\left(q\right)\;\sim \;G_{1}\exp\left(-\frac{q^{2}R_{g,1}^{2}}{3}\right)+B_{1}{\left[erf\left(\frac{qR_{g,1}}{\sqrt{6}}\right)\right]}^{3p_{1}}q^{-p_{1}}\;\exp\left(-\frac{q^{2}R_{g,2}^{2}}{3}\right)+G_{2}\exp\left(-\frac{q^{2}R_{g,2}^{2}}{3}\right)+B_{2}{\left[erf\left(\frac{qR_{g,2}}{\sqrt{6}}\right)\right]}^{3p_{2}}q^{-p_{2}}$$

**Figure 6:**
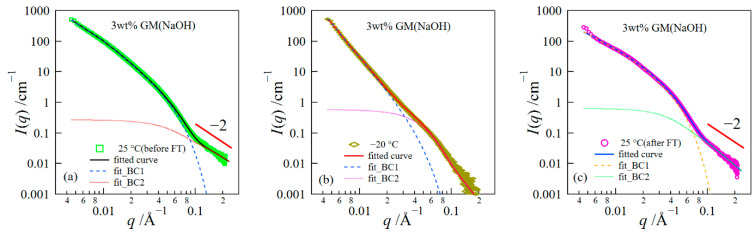
Comparison between SAXS data and fitted curves: (**a**) the structure before freezing, (**b**) the structure during freezing, and (**c**) the structure after thawing.

*G_i_* and *B_i_* (*i* = 1 or 2) denote the Guinier prefactor and a prefactor specific to the power–law scattering, respectively. *R_g_*_,*i*_ and *p_i_* represent the radius of the gyration of a structure at a given level and the exponent, respectively. Figure 6 presents the comparison between the data and the fitted curve in each region. The SAXS data after the thawing was fitted except for the data at *q* < 0.0074 Å^−1^.

**Figure 7:**
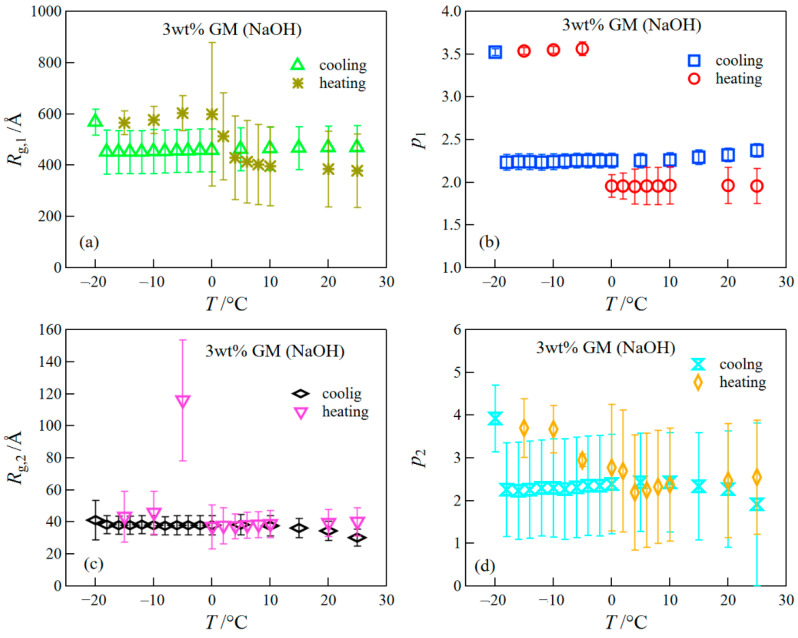
Temperature dependence of parameters (*R_g_*_,1_ (**a**), *p*_1_ (**b**), *R_g_*_,2_ (**c**), and *p*_2_ (**d**)) obtained in the fitting analysis. See Equation (1) about each parameter.

The result of the fitted analysis revealed that the structures before/after the FT treatment were composed of two levels observed at *q*’s lower and higher than ~0.1 Å^−1^, which corresponded to the aggregated structure and the concentration fluctuations inside the aggregated phase, respectively, as mentioned above. We present the result of the parameters obtained in the fitting analysis in Figure 7. The values of *p*_2_ before the freezing were close to 2, although the error showed somewhat large values. The behavior of *q*^−2^ in the high *q* range shows the scattering from random coil chains [35]. When the gel is frozen, *p*_1_ drastically changed and showed values close to ~4. The behavior of *q*^−4^ corresponds to the Porod law, which represents the scattering from a sharp interface [36]; in this case, the scattering behavior comes from the interface scattering of ice crystals. The structure after the thawing did not return to that before the freezing; specifically, the difference in the scattering intensity for the gels before and after the FT treatment was larger in the lower *q*-range. This result suggests that the network structure constructed by the aggregation of GM greatly changed due to the FT treatment. Here, it is important to notice that these experiments were conducted at a slow cooling rate of 1 °C/min. Therefore, the ice crystals should become quite large, as reported by other researchers [12]; as a result, the network structure after thawing became quite distorted.

### 3.3. Influence of Additives on Mechanical and Structural Properties of Freeze-Thawed Gels

**Figure 8:**
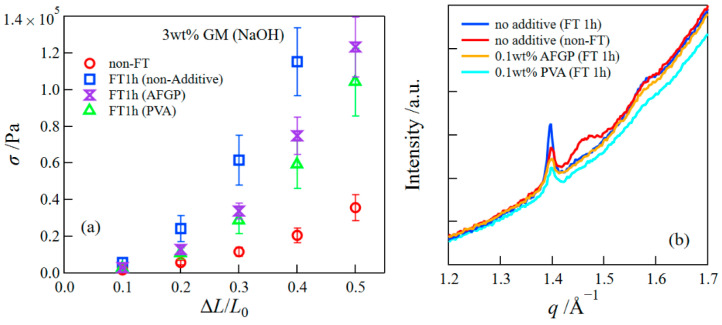
The compressive stress at different strains (**a**) and WAXS curves (**b**) for the freeze-thawed GM/AFGP and GM/PVA gels.

**Figure 9:**
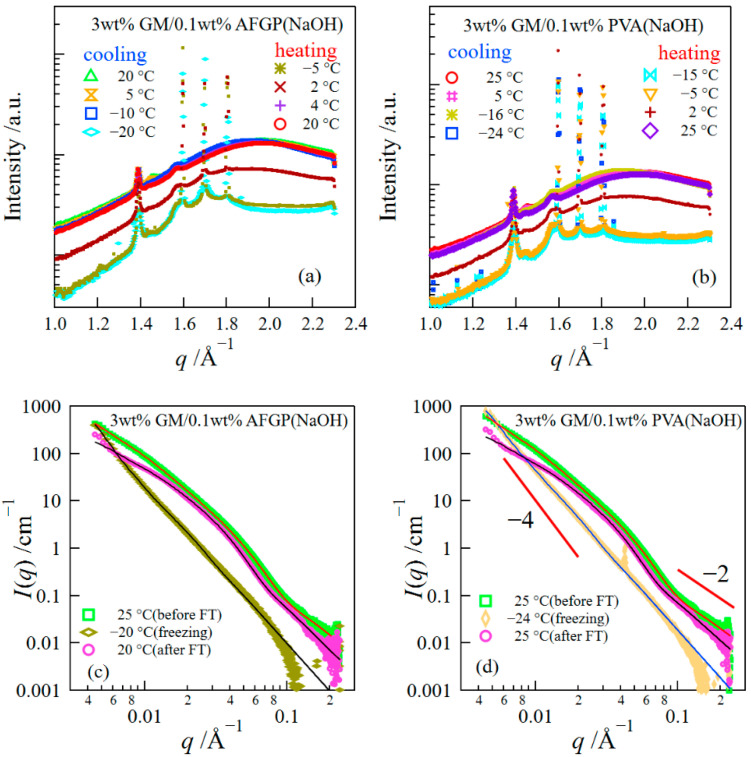
WAXS/SAXS curves for GM/AFGP gels (**a**,**c**) and for GM/PVA (**b**,**d**) gels.

We examined the influence of additives on the mechanical properties of freeze-thawed gels. Figure 8a depicts the compressive stress at different strains for the freeze-thawed GM gels containing 0.1 wt% AFGP or 0.1 wt% PVA. For comparison, the result of GM gels without additives is also shown in the figure. Although the compressive stresses for freeze-thawed GM/AFGP and GM/PVA gels decreased compared with those of the freeze-thawed GM gels, they were higher than those of the GM gel without the FT treatment. Here, we investigated the structures of GM/AFGP and GM/PVA gels. Figure 8b presents WAXS curves for various gels in the range of *q* = 1.2 to 1.7 Å^−1^. The crystalline peak intensity observed at 1.4 Å^−1^ for GM gels without additives increased after the FT treatment, i.e., the FT treatment promoted the crystallization of GM. This behavior is similar to that of freeze-thawed PVA hydrogels, which are well-known as mechanically excellent hydrogels, where the crystallized PVA caused by the FT treatments improves the mechanical performance [8,14,37]. The peak intensities at *q* = 1.4 Å^−1^ for GM/AFGP and GM/PVA gels were lower compared to those of the GM gels with and without the FT treatments, indicating that the addition of AFGP and PVA suppressed the crystallization of GM, which may lower the compressive strength compared with that of freeze-thawed GM gel. Furthermore, the structures of GM/AFGP and GM/PVA gels during the FT process were investigated using simultaneous SAXS/WAXS measurements (Figure 9). The SAXS/WAXS behaviors of both gels were similar to those of the GM gel as shown in Figure 5; the WAXS results show that the hexagonal ice crystals were formed at subzero temperatures, whereas the SAXS behavior could be divided into three regions: (i) the structure before freezing; (ii) the structure during the freezing; and (iii) the structure after thawing. In the region (ii), the scattering behavior in the low *q*-range obeyed the Porod’s law due to the scattering from the sharp interface of ice crystals, whereas in the region (III) the scattering profiles in the very low *q*-range had a slight upturn due to the increase in the inhomogeneity of the gel network arising from the FT treatment. The SAXS profiles for GM/AFGP or GM/PVA hydrogels could also be expressed by the Beaucage equations composed of two structural levels. The result of the parameters obtained in the fitting analysis is shown in Table 1. The parameters obtained for three kinds of gels showed close values. Accordingly, the structures for GM/AFGP and GM/PVA gels before and after the FT process are similar to those of the GM gels without additives, except for the decrease in the amount of GM crystals. Thus, it can be concluded that, although the less amount of GM crystals caused the lower compressive stress for GM/AFGP and GM/PVA gels than that of freeze-thawed GM gels, the stresses became higher compared with those of the GM gel without the FT treatment due to distortion of the network arising from the FT treatment that is unrecoverable after the thawing.

## 4. Conclusions

We investigated the structures of konjac GM gels with and without FT treatment using synchrotron simultaneous SAXS/WAXS techniques. The experiments revealed that the GM gels were constructed by the aggregates and crystallites of GM, whereas the FT treatment promoted the crystallization of GM and the inhomogeneity of the aggregates arising from the ice crystal growth during freezing; as a result, the compressive stress for the freeze-thawed GM gels became greatly higher. Simultaneous synchrotron SAXS/WAXS measurements demonstrated that they were very powerful for examining the structural change of GM gels during the FT process. The structure during the FT process could be classified into three regions: (i) in the region before freezing, the structure was composed of the aggregates of GM and the concentration fluctuations inside them; (ii) during freezing, the hexagonal ice crystals were formed and grew; and (iii) in the region after thawing, the structure did not return to its original form. Additionally, we examined the influence of the additives such as PVA or AFGP on the structural and mechanical properties of freeze-thawed GM gels. Although the addition of PVA or AFGP to the GM gels suppressed the crystallization of GM, it could not prevent the increase in the inhomogeneity of the aggregates due to ice crystal growth; as a consequence, the gel after the FT treatment became moderately stiffer compared with that of GM gels without the FT treatment.

## Figures and Tables

**Table 1:** The result of the fitting analysis.

Sample	*T*/°C	*R_g_*_,1_/Å	*p*_1_	*R_g_*_,2_/Å	*p*_2_
GM	20 (before FT)	470 ± 84	2.3 ± 0.1	35 ± 6	2.3 ± 1.4
	25 (after FT)	379 ± 143	2.0 ± 0.2	40 ± 9	2.6 ± 1.3
GM/PVA	25 (before FT)	480 ± 81	2.4 ± 0.1	32 ± 7	2.2 ± 1.3
	25 (after FT)	377 ± 144	2.0 ± 0.2	47 ± 10	2.7 ± 0.8
GM/AFGP	25 (before FT)	477 ± 94	2.4 ± 0.1	32 ± 9	2.4 ± 1.5
	20 (after FT)	383 ± 173	2.0 ± 0.2	49 ± 15	2.9 ± 0.9

## Data Availability

Data are contained within the article.
